# Inhibiting USP14 ameliorates inflammatory responses in trophoblast cells by suppressing MAPK/NF‐κB signaling

**DOI:** 10.1002/iid3.465

**Published:** 2021-06-05

**Authors:** Yingzi Zhao, Fang Zong

**Affiliations:** ^1^ Department 3 of Obstetrics Cangzhou Central Hospital Cangzhou Hebei China

**Keywords:** NF‐κB, preeclampsia, proinflammatory cytokine, UPS14

## Abstract

**Background:**

Preeclampsia can cause severe consequences for pregnant women and infants, and developing effective medicine or methods to prevent or treat patients with preeclampsia is urgently needed. Ubiquitin‐specific protease 14 (USP14) has emerged as a critical regulator in the development of human cancers and neurodegenerative diseases. However, its role in preeclampsia remains elusive.

**Methods:**

The expression of USP14 in placental tissues from healthy donors and preeclampsia patients were determined by quantitative reverse transcription PCR assay. The protein levels of targeted genes were evaluated by Western blotting assay. Small interfering RNA‐mediated gene knockdown was used to reduce USP14 expression in trophoblast cell lines.

**Results:**

The expression levels of USP14 and proinflammatory cytokine were substantially upregulated in placental tissues from preeclampsia patients. Knockdown or inhibition of USP14 significantly abrogated hypoxia/reoxygenation‐induced upregulation of nuclear factor kappa B (NF‐κB) activation and proinflammatory cytokine production.

**Conclusion:**

Our results suggested that USP14 promotes proinflammatory cytokine production through activation of NF‐κB. Developing drugs targeting USP14 may be beneficial for the prevention or treatment of patients with preeclampsia.

## Graphical abstract

(1) The expression levels of USP14 and proinflammatory cytokine were substantially upregulated in placental tissues. (2) Developing drugs targeting USP14 may be beneficial for the prevention or treatment of patients with preeclampsia.

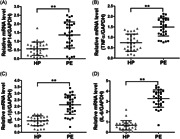

## 1 INTRODUCTION

Preeclampsia is one of the most common maternal and perinatal complications during pregnancy, which can cause serious damages to tissues and organs, leading to impairing liver and kidney function, blood clotting, seizures, pulmonary edema, and even maternal or infant morbidity and mortality.^1^, ^2^, ^3^ The incidence of preeclampsia is around 2%–8% among pregnancies and more prevalent in the middle‐ or low‐income countries.^4^ The occurrence of preeclampsia is positively associated with obesity and cardiovascular diseases.^5^ The detailed mechanism of preeclampsia is not fully understood. It is believed that dysregulation of extravillous cytotrophoblasts invasion and spiral artery transformation, resulting in insufficient uteroplacental blood flow. These conditions cause focal decidual hypoxia, leading to an increase in reactive oxygen species or a lack of ATP. The upregulated placental oxidative stress, in turn, leads to the excessive release of inflammatory mediators, causing pregnancy complications such as preeclampsia.^6^, ^7^ The treatment for preeclampsia is not very effective, especially for those in early pregnancy. Oral or intravenous (IV) medication interventions may be given to pregnant women to lower blood pressure, but they may impose adverse effects on maternities and perinatal infants.^5^, ^8^ Thus, it is highly important to elucidate the molecular mechanism contributing to the development of preeclampsia.

Ubiquitin‐specific proteases (USPs), a member of deubiquitinases (DUBs), are able to rescue proteins from degradation by removing ubiquitin from ubiquitinated proteins.^9^ Accumulating evidence suggests that USPs play critical roles in the regulation of multicellular processes, including autophagy, mitophagy, innate immunity, and oncogenesis.^10^, ^11^, ^12^ USP14 is one of the regulators of proteasomal degradation and has been implied to be involved in neurodegenerative disorders and multiple tumor development and progression.^11^ For example, USP14 directly influences autophagy and mitophagy in neurons and ultimately affects age‐related neurodegenerative disorders.^13^ IU1, an inhibitor of USP14, can increase UPS activities, promote Tau degradation, and enhance mitochondrial elimination in neuronal cells.^14^, ^15^, ^16^ Similarly, dysregulation of USP14 has been reported in several tumors, including breast cancer, lung adenocarcinoma, and epithelial ovarian cancer.^17^, ^18^, ^19^ However, whether USP14 is involved in preeclampsia remains elusive.

In this study, we aimed to investigate the expression profile of USP14 in placental tissues from healthy donors and preeclampsia patients and to explore the potential molecular mechanism and functional role of USP14 in preeclampsia using placental trophoblast cell lines treated with hypoxia/reoxygenation (H/R) as in vitro cell model for preeclampsia studying. The findings of this study may provide novel insight into USP14 in the regulation of preeclampsia.

## 2 MATERIALS AND METHODS

### 2.1 Cell culture and treatment

Both B6Tert‐1 and HTR‐8/Svneo used in this study are immortalized human cytotrophoblast cell lines. The human trophoblast cell line B6Tert‐1 and human chorionic trophoblast cell line HTR‐8/SVneo were purchased from American Type Culture Collection. Cells were cultured in RPMI‐1640 medium containing 10% fetal bovine serum (Gibco) and were maintained in a 37° incubator with 5% CO_2_. For H/R treatment, B6Tert‐1 or HTR‐8/SVneo cells were initially cultured in hypoxia condition (2% O_2_) for 8 h and then cultured in normal condition (20% O_2_) for another 16 h. Lentivirus carrying sh‐negative control (NC), shUSP14#1 (LPP‐HSH022063‐LVRH1MH‐050), and shUSP14#2 (LPP‐HSE022063‐LVE003‐050) were obtained from GenScript. Cells were transduced with lentiviral particle at multiplicity of infection at 10. IU1 (I1911‐5MG) was purchased from Millipore Sigma.

### 2.2 Human samples

Human placental tissues from healthy donors (*n* = 30) and preeclampsia patients (*n* = 30) were collected from Cangzhou Central Hospital. Written informed consent was obtained from all participants, and this study was approved by the Ethic Committee of Cangzhou Central Hospital.

### 2.3 Quantitative reverse transcription PCR (RT‐qPCR)

Cells were washed and spun down to collected cell pellets. The human tissues were cut into small pieces. Trizol reagent (Invitrogen) was applied to extract total RNA. SuperScript IV Reverse transcriptase (Invitrogen) was used to produce complementary DNA. TransStart® Green qPCR SuperMix (TransGen Biotech) was used to perform qRT‐PCR. Primers used were as follows: USP14: F: 5′‐GGCTTCAGCGCAGTATATTA‐3′, R: 5′‐CAGATGAGG AGTCTGTCTCT‐3′;

Tumor necrosis factor‐α (TNF‐α)—F: 5′‐GTCACTCATTGCTGAGCCTCT‐3′, R: 5′‐AGCTTCTTCCCACCCACAAG‐3′; interleukin‐6 (IL‐6)—F: 5′‐CATCACCATCTTCCAGGAG‐3′, R: 5′‐AGGCTGTTGTCATACTTCTC‐3′; IL‐1β—F: 5′‐ACAGTGGCAATGAGGATG‐3′, R: 5′‐TGTAGTGGTGGTCGGAGA‐3′; glyceraldehyde 3‐phosphate dehydrogenase (GAPDH)—F: 5'‐GCACCGTCAAGGCTGAGAAC‐3', R: 5'‐TGGTGAAGACGCCAGTGGA‐3′.

### 2.4 Detection of cytokines

To assess the expression of TNF‐α, IL‐1β, IL‐6, and IL‐10, cells were seeded in 12‐well plates, and the cell culture supernants of HTR8/Svneo cells treated with dimethyl sulfoxide (DMSO) or 25 μM IU1 under H/R conditions were collected for cytokines' detection. The protein expression levels of TNF‐α, IL‐1β, IL‐6, and IL‐10 were quantified by using specific human ELISA assay kits (Thermo Fisher Scientific) following the manufacturer's protocols.

### 2.5 Western blotting

Total proteins from cells were extracted using RIPA lysis buffer with protease inhibitor cocktail (MyBioSource). Protein concentration was determined by bicinchoninic acid analytical method. A total of 30 μg randomly chose protein samples from each group were separated by sodium dodecyl sulphate–polyacrylamide gel electrophoresis using an 8% precast gel (Thermo Fisher Scientific). The proteins were transferred to a polyvinylidene difluoride membrane (Bio‐Rad) and blocked with 5% nonfat milk. The membrane was immersed in primary antibody solution overnight and then incubated with secondary antibody. The protein signal was developed using ECL reagent and visualized using iBright™ FL1500 Imaging System (Thermo Fisher Scientific). Anti‐USP14 (#11931S), Anti‐p‐p65 (#3033S), Anti‐p65 (#8242S), Anti‐p‐p38 (#4511S), Anti‐p38 (#8692S), Anti‐GAPDH (#5174S) were purchased from Cell Signaling Technology.

### 2.6 Statistical analysis

All data were presented as mean ± *SD*. The Student's *t* test was applied to assess the difference between the two groups, and one‐way analysis of variance analysis with a post hoc test was applied to assess the difference between multiple groups. Data analysis was performed on Prism 6.0 software. *p* < 0.05 was considered as a statistically significant difference.

## 3 RESULTS

### 3.1 USP14 and proinflammatory cytokines were upregulated in placental tissues from preeclampsia patients

**Figure 1:**
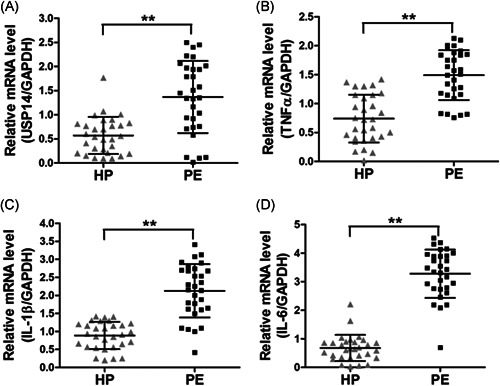
USP14 and proinflammatory factors are elevated in preeclampsia patients. The mRNA levels of USP14 (A), TNF‐α (B), IL‐6 (C), and IL‐1β (D) in healthy pregnant women (HP, *n* = 30) and preeclampsia (PE) patients (*n* = 30) were measured by RT‐qPCR. **p < 0.01. IL, interleukin; mRNA, messenger RNA; TNF‐α, tumor necrosis factor‐α; USP14, Ubiquitin‐specific proteases 14

To investigate the potential relevance of USP14 in preeclampsia, we collected placental tissues from healthy donors (*n* = 30) and preeclampsia patients (*n* = 30). The messenger RNA (mRNA) levels of USP14 in these placental tissues were determined by RT‐qPCR, and the results showed that the expression levels of USP14 were significantly higher in placental tissues from preeclampsia patients than those from healthy donors (Figure 1A). A previous study suggested that USP14 can regulate proinflammatory cytokine expression in THP‐1 cells. We also tested the mRNA levels of proinflammatory cytokines (TNF‐α, IL‐6, and IL‐1β) in those placental tissues. As depicted in Figure 1B–D, dramatic upregulation of TNF‐α, IL‐6, and IL‐1β was observed in placental tissues from preeclampsia patients compared to healthy donors.

### 3.2 USP14 and p‐p65 were elevated in placental tissues from preeclampsia patients

**Figure 2:**
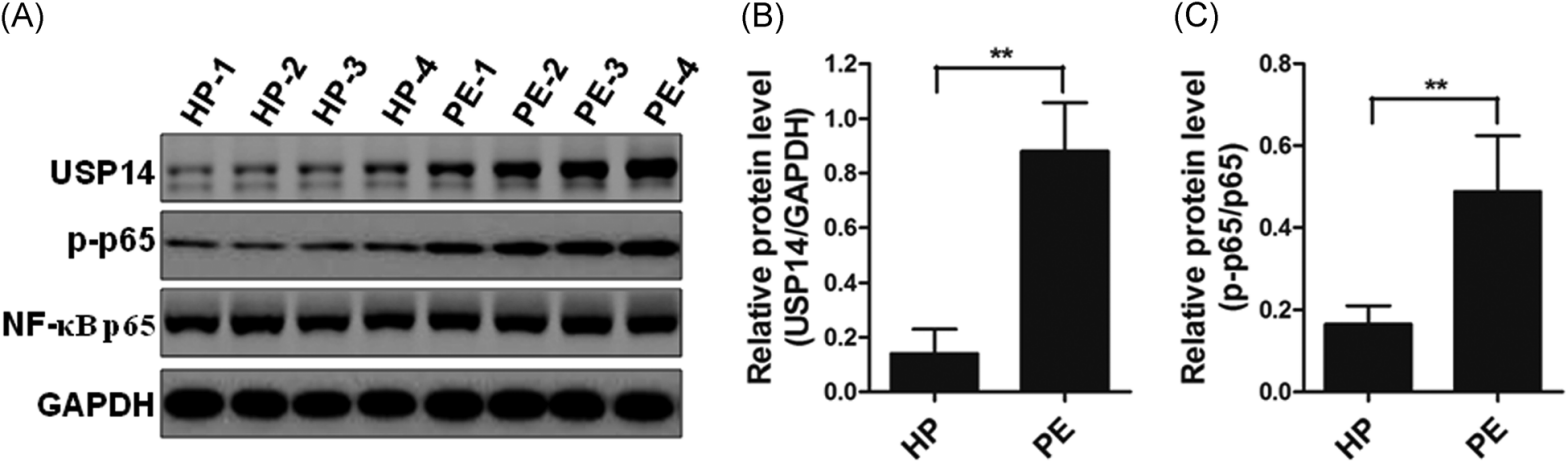
The protein levels of USP14 and phosphorylated NF‐κB p65 are upregulated in preeclampsia patients. Western blotting was performed to detect the protein levels of USP14, p‐p65, and NF‐κB p65 in HP and PE patients. The representative blots were as shown in (A), and relative optical density was measured (B and C). **p < 0.01. HP, healthy pregnant women; NF‐κB, nuclear factor kappa B; PE, preeclampsia; Ubiquitin‐specific proteases 14

It was reported that USP14 regulates lipopolysaccharides‐induced proinflammatory cytokine expression through mediating nuclear factor kappa B (NF‐κB) activity.^20^ To further confirm this result, we assessed the protein expression of USP14, p‐p65, and total‐p65 in placental tissues from healthy donors (*n* = 4) and preeclampsia patients (*n* = 4). As illustrated in Figure 2A–C, the expression levels of USP14 and p‐p65, but not total‐p65, were substantially increased in preeclampsia patients' placental tissues compared to healthy donors' placental tissues.

### 3.3 H/R treatment induced upregulation of USP14 and p‐p65 in trophoblast cell lines

**Figure 3:**
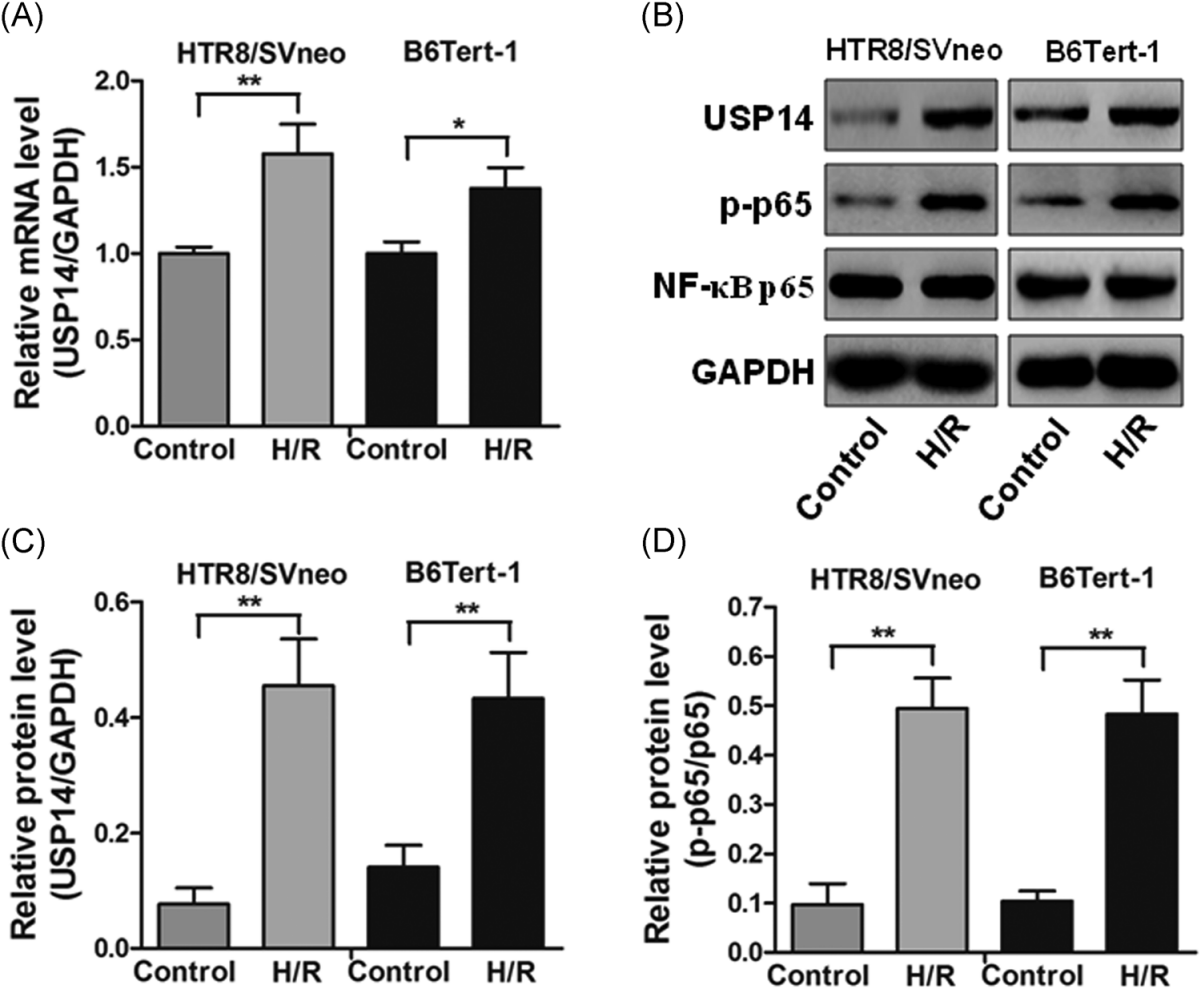
USP14 and phosphorylated NF‐κB p65 are upregulated in trophoblast cells under H/R conditions. (A) The mRNA levels of USP14 in HTR8/Svneo and B6Tert‐1 cells under H/R conditions were measured by RT‐qPCR. (B) The protein levels of USP14, p‐p65, and NF‐κB p65 in HTR8/Svneo and B6Tert‐1 cells under H/R conditions were determined by Western blotting. (C and D) The relative optical density of these genes was measured. *p < 0.05, **p < 0.01. H/R, hypoxia/reoxygenation; mRNA, messenger RNA; NF‐κB, nuclear factor kappa B; RT‐qPCR, quantitative reverse transcription PCR; TNF‐α, tumor necrosis factor‐α; USP14, Ubiquitin‐specific proteases 14

To explore the potential molecular mechanism involved in preeclampsia, the two placental trophoblast cell lines (HTR8/Svneo and B6Tert‐1) were subjected to H/R treatment to mimic the preeclampsia condition. H/R treatment substantially enhanced the expression levels of USP14 mRNA and protein expression (Figure 3A–C), and p‐p65 protein expression (Figures 3B and 3D) in HTR8/Svneo and B6Tert‐1 cell lines.

### 3.4 Depletion of USP14 abrogated H/R‐induced upregulation of USP14, p‐p65, and proinflammatory cytokines

**Figure 4:**
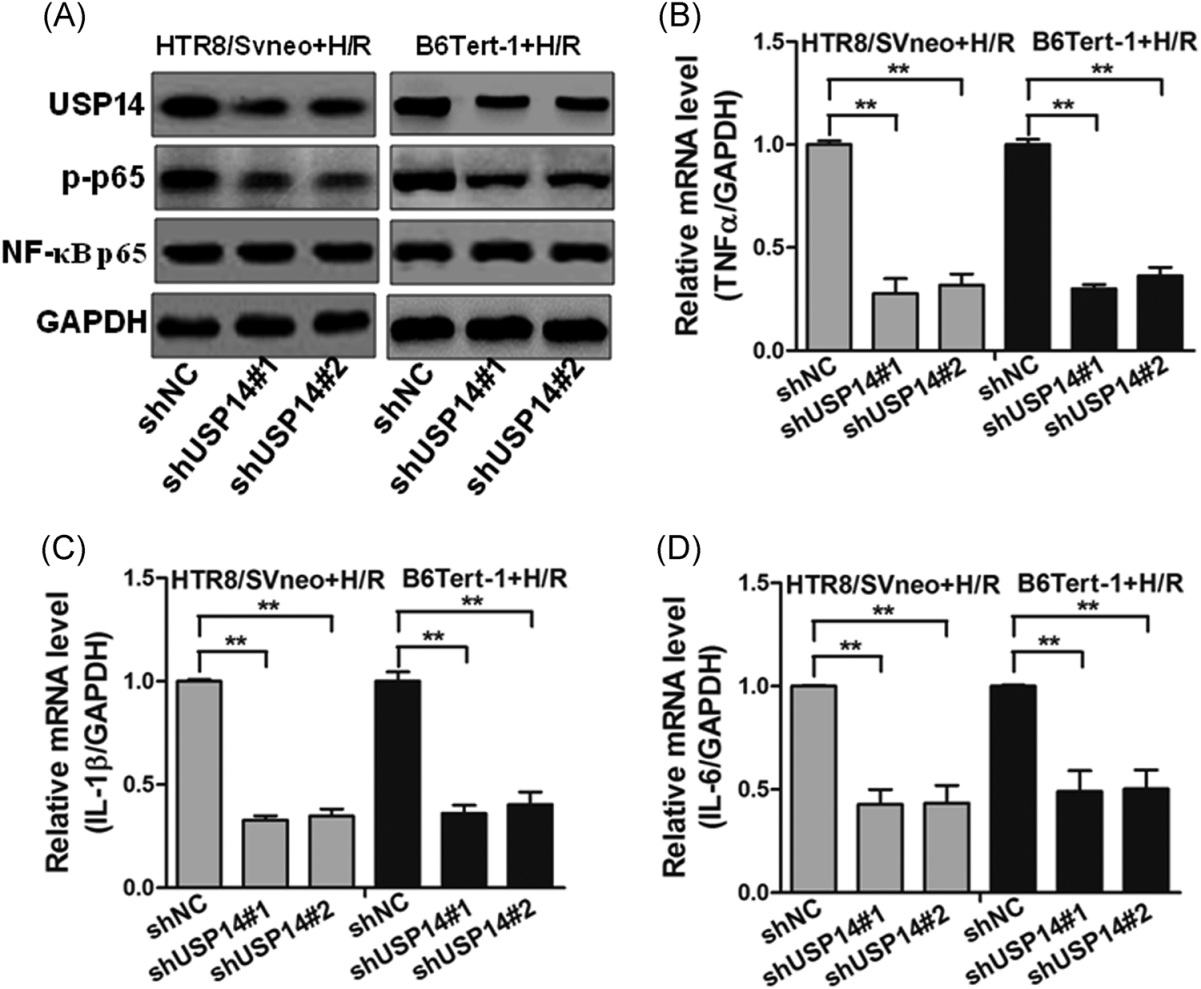
Silence of USP14 by shRNAs inhibits the expression levels of proinflammatory factors in trophoblast cells under H/R conditions. (A) Western blotting was applied to detect the protein levels of USP14, p‐p65, and NF‐κB p65 in HTR8/Svneo and B6Tert‐1 cells infected with shUSP14‐derived lentivirus under H/R conditions. (B–D) The mRNA levels of TNF‐α (B), IL‐1β (C), and IL‐6 (D) were measured by RT‐qPCR in HTR8/Svneo and B6Tert‐1 cells infected with shUSP14‐derived lentivirus under H/R conditions. **p < 0.01. H/R, hypoxia/reoxygenation; IL, interleukin; mRNA, messenger RNA; siRNA, small interfering RNA; TNF‐α, tumor necrosis factor‐α; USP14, Ubiquitin‐specific proteases 14

To further study the potential role of USP14 in preeclampsia, we aimed to knockdown of USP14 in trophoblast cell lines. HTR8/Svneo and B6Tert‐1 were transduced with lentivirus containing sh‐NC or two shRNA targeting USP14 (shUSP14#1 and shUSP14#2). As showed in Figure 4A, overexpression of shUSP14#1 or shUSP14#2 resulted in substantially decreased USP14 and p‐p65 expression in HTR8/Svneo and B6Tert‐1 cells. Importantly, we found that depletion of USP14 resulted in downregulation of TNF‐α, IL‐6, and IL‐1β mRNA expression in HTR8/Svneo and B6Tert‐1 (Figure 4B–D), suggesting that USP14 regulates TNF‐α, IL‐6, and IL‐1β expression through activation of NF‐κB activity.

### 3.5 Inhibition of USP14 suppressed H/R‐induced NF‐κB and MAPK activation

**Figure 5:**
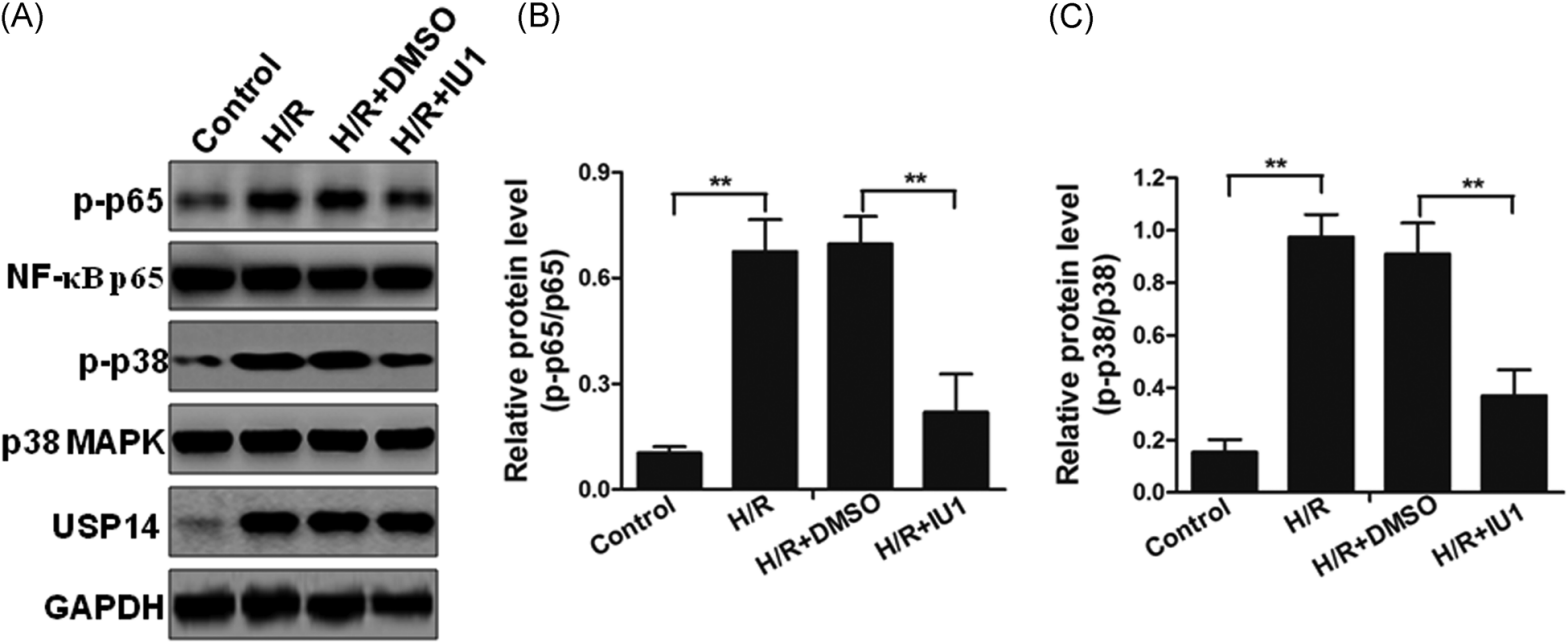
IU1 inhibits H/R‐induced NF‐κB and MAPK activation in trophoblast cells. (A) Western blotting was used to detect the protein levels of p‐p65, NF‐κB p65, p‐p38, p38 MAPK, and USP14 in HTR8/Svneo cells treated with DMSO or 25 μM IU1 under H/R conditions. (B and C) The relative optical density of these genes was measured. **p < 0.01. DMSO, dimethyl sulfoxide; H/R, hypoxia/reoxygenation; NF‐κB, nuclear factor kappa B

To investigate the role of USP14 in H/R‐induced NF‐κB and MAPK activation, HTR8/Svneo cells were pretreated with or without IU1, a USP14 inhibitor, or DMSO as a control, and then subjected to H/R treatment. We observed that H/R treatment significantly promoted upregulation of p‐p65 and p‐p38 in nontreated or DMSO‐treated groups (Figure 5A–C). However, IU1 pretreatment abolished H/R‐induced upregulation of p‐p65 and p‐p38 in HTR8/Svneo cells, implying that USP14 plays a pivotal role in H/R‐promoted NF‐κB and MAPK activation.

### 3.6 Inhibition of USP14 reduced H/R‐induced proinflammatory cytokine production

**Figure 6:**
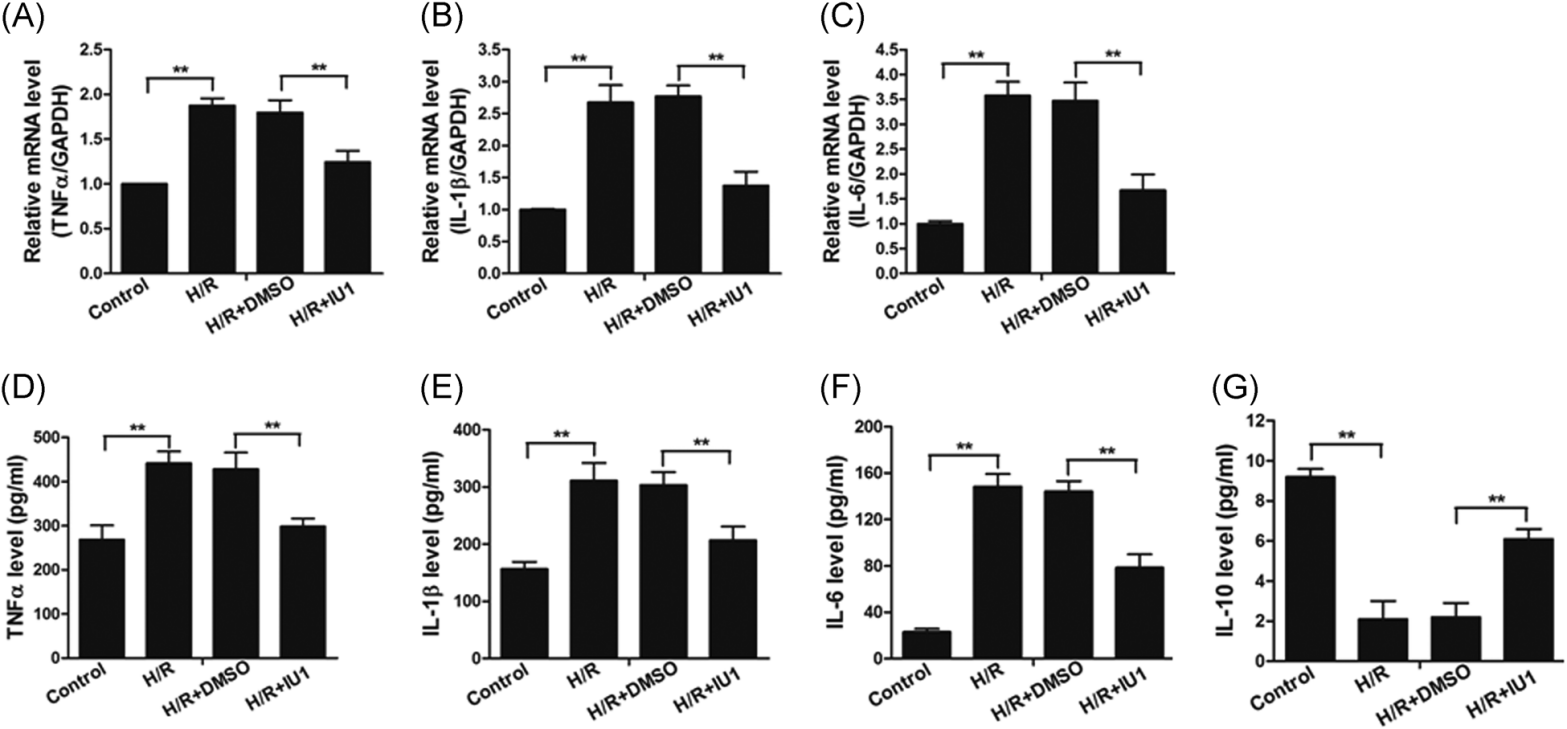
IU1 inhibits the expression of proinflammatory factors in trophoblast cells under H/R conditions. (A–C) The mRNA levels of TNF‐α (A), IL‐1β (B) and IL‐6 (C) were measured by RT‐qPCR in HTR8/Svneo cells treated with DMSO or 25 μM IU1 under H/R conditions. (D–G) The expression levels of TNF‐α (D), IL‐1β (E), IL‐6 (F), and IL‐10 (G) in cell culture supernants were measured by ELISA in HTR8/Svneo cells treated with DMSO or 25 μM IU1 under H/R conditions. **p < 0.01. DMSO, dimethyl sulfoxide; ELISA, enzyme‐linked immunosorbent assay; H/R, hypoxia/reoxygenation; IL, interleukin; mRNA, messenger RNA; NF‐κB, nuclear factor kappa B; RT‐qPCR, quantitative reverse transcription PCR; TNF‐α, tumor necrosis factor‐α; USP14, Ubiquitin‐specific proteases 14

Finally, to study the effect of USP14 in H/R‐promoted proinflammatory cytokine expression, HTR8/Svneo cells were pretreated with or without IU1 or DMSO, and subjected to H/R stimulation. We measured the expression levels of TNF‐α, IL‐6, and IL‐1β in these cells. We found that H/R stimulation significantly induced TNF‐α, IL‐6, and IL‐1β mRNA and protein expression, and this promotion effect was abrogated by IU1 pretreatment (Figure 6A–F), suggesting that USP14 is essential for H/R‐induced proinflammatory cytokine expression. In addition, the expression of IL‐10 was significantly inhibited by H/R stimulation, and this inhibitory effect was abrogated by IU1 pretreatment (Figure 6G).

## 4 DISCUSSION

Preeclampsia is defined as new‐onset hypertension in pregnancy after 20 weeks along with proteinuria. Without appropriate treatment, preeclampsia can be life‐threatening to pregnant women and infants.^2^, ^3^ The saying “prevention is the best treatment” holds true, and changing life style may be beneficial in the prevention of preeclampsia. Pregnant women with a high risk of preeclampsia are recommended to take sufficient rest and proper exercise and have a healthy diet with high protein and reduced salt. Some dietary supplementations (e.g., calcium, antioxidants, and vitamins) and antiplatelet drugs (e.g., low dose aspirin) may also reduce the risk of preeclampsia.^21^, ^22^ Some Chinese herbal medicines attract increasing attention on alleviation or prevention of preeclampsia, but none are proved in randomized clinical trials.^23^ When hypertension causes direct vascular damage, resulting in life‐threatening complications. The only two kinds of available drugs for preeclampsia treatment are antihypertensive agents, aiming to reduce blood pressure, and anticonvulsant (e.g., magnesium sulfate).^24^, ^25^ Identifying key genes in the placenta that contribute to the pathophysiology of preeclampsia is another promising method for developing alternative strategies for the prevention and treatment of eclampsia.

Accumulating evidence has demonstrated that USP14 is involved in the occurrence and development of various human cancers. USP14 is reported to be overexpressed in multiple tumor types and is closely associated with tumor recurrence and poor prognosis.^10^, ^18^, ^19^ For example, Wu et al.^26^ reported that USP14 expression levels were significantly upregulated in lung adenocarcinoma cell lines and nonsmall cell lung cancer (NSCLC) tissues, and high USP14 levels were correlated with the shorter overall survival rate of patients with lung adenocarcinoma.^26^ Similarly, overexpression of cytoplasmic USP14 was confirmed in colorectal cancer tissues and is associated with lymph node metastasis and worse overall survival.^27^ Besides the USP14 oncogenic role in human cancers, its role in neurodegenerative disorders has been extensively investigated. Earlier studies of USP14 using mice model showed that mice with USP14 depletion or inactivation exhibited abnormalities in neuromuscular junction function and developmental defects in motor neurons.^28^ Interestingly, overactivation of DUBs may contribute to the progression of Parkinson's disease.^29^ Thus, inhibition of DUBs using inhibitors (e.g., IU1, a USP14 inhibitor) may yield beneficial effects against the progression of Parkinson's disease. We revealed that USP14 is significantly upregulated in placental tissues from patients with preeclampsia, suggesting USP14 may play a crucial role in the development or progression of preeclampsia.

We hypothesized that USP14 might influence preeclampsia progression by mediating the inflammatory response. Preeclampsia is associated with immune activation, as often presented by enhanced production of proinflammatory cytokines (e.g., TNF‐α, IL‐6, and IL‐1β) and reduced secretion of antiinflammatory cytokines (e.g., IL‐10 and IL‐4).^30^ The dysregulation of the inflammatory response is generally induced by placental ischemia, which contributes to the overall pathophysiology associated with preeclampsia.^31^ Indeed, similar to USP14, markedly upregulation of p‐p65, TNF‐α, IL‐6, and IL‐1β was observed in preeclampsia patients' placental tissues compared to healthy placental tissues, implying that upregulation of USP14 may affect proinflammatory cytokine expression in placenta through affecting NF‐κB activation. The potential role of UPS14 in the regulation of inflammation has been depicted in several publications. Li et al.^32^ showed that USP14 regulates IκBα deubiquitination and degradation to promote NF‐κB activation in chondrocytes. Min et al.^20^ reported that USP14 deubiquitinates TAK1‐binding protein 2 protein to active NF‐κB signaling, leading to enhance the production of proinflammatory cytokines.^20^ Consistent with these findings, we also found that knockdown of USP14 significantly inhibited H/R‐induced upregulation of USP14 and p‐p65 expression. Similarly, inhibit USP14 activity using IU1 yielded suppression of p‐p65 and p‐p38 in trophoblast cells. These results supported our conclusion that USP14 regulates proinflammatory cytokine production through mediating NF‐κB activation in placenta. Although the findings are exciting and encouraging, there are still some questions that deserved further investigation. For instance, how USP14 regulates MAPK/NF‐κB signaling pathways? It also would be interesting in the future if we can find small molecules function as USP14 inhibitor, and to test its potential as a drug candidate to prevent PE occurrence.

## 5 CONCLUSION

This study, for the first time, revealed the potential role of USP14 in preeclampsia progression. Our data implied that placental ischemia promotes upregulation of USP14, and the latter enhances the production of proinflammatory cytokines via activation of NF‐κB signaling, leading to worsening preeclampsia progression. Thus, USP14 may serve as an excellent therapeutic candidate for developing strategies for preeclampsia prevention and management.

## CONFLICT OF INTERESTS

The authors declare that there are no conflict of interests.

## ETHICS STATEMENT

This study was approved by the Ethic Committee of Cangzhou Central Hospital. All procedures performed in studies involving human participants were in accordance with the ethical standards of the institutional and/or national research committee and with the 1964 Helsinki declaration and its later amendments or comparable ethical standards.

## Data Availability

Data could be obtained upon request to the corresponding author.
